# Robotic Pancreaticoduodenectomy: Current Evidence and Future Perspectives

**DOI:** 10.3390/jcm14238372

**Published:** 2025-11-25

**Authors:** Silvio Caringi, Antonella Delvecchio, Annachiara Casella, Cataldo De Palma, Valentina Ferraro, Rosalinda Filippo, Matteo Stasi, Nunzio Tralli, Tommaso Maria Manzia, Riccardo Memeo, Michele Tedeschi

**Affiliations:** 1 Unit of Hepato-Biliary and Pancreatic Surgery, “F. Miulli” General Hospital, Acquaviva delle Fonti, 70021 Bari, Italy; a.delvecchio@miulli.it (A.D.); a.casella@miulli.it (A.C.); v.ferraro@miulli.it (V.F.); r.filippo@miulli.it (R.F.); matteo.stasi@miulli.it (M.S.); n.tralli@miulli.it (N.T.); r.memeo@miulli.it (R.M.); m.tedeschi@miulli.it (M.T.); 2 Department of Surgery, Università Degli Studi Roma “Tor Vergata”, Via Montpellier 1, 00133 Rome, Italy; 3 Department of Medicine and Surgery, LUM University, 70010 Casamassima, Italy; 4 Pancreatic Surgery, IRCCS Humanitas Research Hospital, 20089 Rozzano, Italy; cataldo.depalma@st.hunimed.eu; 5 Transplant and HPB Unit, Department of Surgery Sciences, University of Rome Tor Vergata, 00133 Rome, Italy; manzia@med.uniroma2.it

**Keywords:** robotic pancreaticoduodenectomy, minimally invasive surgery, pancreatic cancer surgery

## Abstract

Background: Robotic pancreaticoduodenectomy (RPD) is a less invasive alternative to open pancreaticoduodenectomy (OPD) with the potential for perioperative advantage. Concerns remain regarding its technical difficulty, cost, and oncologic adequacy. Methods: Review of PubMed, MEDLINE, Scopus, and Embase databases was conducted (January 2000–October 2025), focusing on systematic reviews, meta-analyses, and significant comparative studies of RPD. Outcomes assessed were perioperative outcomes, oncologic sufficiency, learning curve, model training, cost-effectiveness, and future developments. Results: Several studies report comparable R0 rates and lymph node yield between RPD and OPD, with reduced blood loss, shorter postoperative hospital stay, and faster recovery in high-volume centers. Morbidity (35–50%) and 90-day mortality (<2%) are similar to open or laparoscopic surgery. Competence is usually achieved after 40–60 cases, while optimal outcomes are achieved after 80–100 procedures. Structured mentorship and simulation training improve safety and reproducibility. Novel technologies such as augmented reality, intraoperative fluorescence, and artificial intelligence-based navigation may also enhance accuracy and shorten the learning curve. Conclusions: RPD appears to be a safe and effective minimally invasive option in carefully selected patients if done in specialized, high-volume centers. Future studies need to resolve long-term oncologic results, cost-effectiveness, and the role of next-generation robotic systems.

## 1. Introduction

Pancreaticoduodenectomy (PD) remains among the most demanding procedures in hepatopancreatobiliary (HPB) surgery, due to the complex anatomy of the head of the pancreas, requirement for various anastomoses, and potential for high perioperative morbidity and mortality [1,2]. Advances in surgical technique, perioperative management, and centralization of services have reduced mortality and improved outcomes over the past decades; nevertheless, PD morbidity remains significant even within high-volume institutions [1,3].

The advent of minimally invasive surgery (MIS) has transformed a vast majority of abdominal surgeries with the promise of reduced blood loss, reduced hospitalization, and faster recovery. In HPB surgery, the introduction of laparoscopic PD (LPD) in the mid-1990s was a landmark development [1]. However, the uptake of LPD has remained low, largely due to technical inconvenience in replicating the open technique’s complex sequence of resection and reconstruction by stiff laparoscopic instruments, two-dimensional imaging, fulcrum effects, and problematic ergonomics [3].

In this regard, robotic surgical systems are an effective next-generation alternative platform for advanced minimally invasive HPB surgery. With three-dimensional stereoscopic visualization, articulated instruments with greater degrees of freedom, tremor-filtration, and improved operator ergonomics, robotic platforms in theory transcend most of the limitations related to laparoscopy [2,4]. In the realm of PD, robotic pancreaticoduodenectomy (RPD) uptake has picked up pace over the past few years in specialist units with initial reports demonstrating feasibility and safety in appropriately selected patients [2,5].

Nevertheless, despite its universal acceptance, several important questions have remained unanswered. They include the size of benefit with improved outcomes compared with open or laparoscopic PD (in terms of morbidity, adequacy for oncology, and long-term survival), cost-effectiveness of the robotic method across different healthcare systems, learning curve, and volume required at the institution for safe implementation, and standardization of the technique across different environments. So far, evidence has largely been based on retrospective series, often from high-volume centers, whose generality is questionable [6].

The purpose of the current narrative review is to provide an overview and state-of-the-art appraisal of the evidence on RPD. We will address its evolution in history, technical considerations, perioperative and oncologic outcomes, learning curve and centralization issues, and costs and economic considerations, as well as limitations of the available evidence and directions for future research and practice. In so doing, we intend to offer a resource for clinicians, surgeons, and researchers who are considering or undergoing RPD, and elucidate the place of this procedure in modern HPB practice.

## 2. Methods

Although this is a narrative work and not a systematic review, methodological transparency was preserved to enhance reproducibility and reduce selection bias. The review followed SANRA (Scale for the Assessment of Narrative Review Articles) guidelines, which provide a structured system for evaluating the quality of non-systematic reviews [7].

### 2.1. Search Strategy

A comprehensive literature search of PubMed/MEDLINE, Scopus, and Embase databases was conducted for the duration from January 2000 to October 2025. The search employed a combination of Medical Subject Headings (MeSH) and free-text terms for robotic pancreaticoduodenectomy as follows: “robotic pancreaticoduodenectomy”, “robot-assisted Whipple”, “robotic pancreatic surgery”, “minimally invasive pancreaticoduodenectomy”, “robotic pancreatoduodenectomy”, and “robotic pancreatic resection”.Boolean operators and filters were applied as follows: ([“robotic” OR “robot-assisted”]) AND ([“pancreaticoduodenectomy” OR “Whipple procedure”]) AND ([“outcomes” OR “safety” OR “oncologic results” OR “cost-effectiveness”]).

The search was limited to English-language human studies. Additional articles were identified by manual cross-referencing of bibliographies of relevant reviews, meta-analyses, and large cohort studies [8].

Duplicates were removed using EndNote (fields matched: title, DOI, first author, year), supplemented by manual cross-checking.

Full copy-paste Boolean search strings were documented for reproducibility and are reported as follows:

- PubMed search string (last search 15 October 2025, via PubMed.gov):

(“robotic”[Title/Abstract] OR “robot-assisted”[Title/Abstract]) AND (“pancreaticoduodenectomy”[MeSH Terms] OR “Whipple procedure”[Title/Abstract]) AND (“outcomes”[Title/Abstract] OR “safety”[Title/Abstract] OR “oncologic results”[Title/Abstract] OR “cost-effectiveness”[Title/Abstract]).

- Embase search string (Ovid, last search 15 October 2025):

(‘robotic pancreaticoduodenectomy’:ab,ti OR ‘robot-assisted Whipple’:ab,ti) AND (‘outcomes’ OR ‘safety’ OR ‘oncologic*’).

- Scopus search string (Elsevier, last search 15 October 2025):

TITLE-ABS-KEY (robotic OR robot-assisted) AND TITLE-ABS-KEY (pancreaticoduodenectomy OR Whipple)

### 2.2. Eligibility Criteria

Studies were included if they met the following criteria:

Inclusion criteria:

1. Reported clinical outcomes of RPD for pancreatic and periampullary benign or malignant disease;
2. Comparative RPD vs. open or laparoscopic pancreaticoduodenectomy (OPD/LPD) studies;
3. Systematic reviews, meta-analyses, or large retrospective/prospective series (> 20 patients);
4. Peer-reviewed journal-published articles.

Exclusion criteria:

1. Case reports or small case series (<10 patients);
2. Experimental or pre-clinical studies with minimal clinical data;
3. Non-English publications or conference abstracts with no full-text data.

### 2.3. Data Extraction and Synthesis

From every study included, data were taken on study design, sample size, patient demographics, operative details (operative time, blood loss, conversion rate), postoperative outcomes (morbidity, mortality, postoperative pancreatic fistula (POPF), delayed gastric emptying (DGE), post-pancreatectomy hemorrage (PPH)), oncologic outcomes (R0 resection, lymph node yield, and survival), and cost-effectiveness measures. Data were summarized qualitatively due to heterogeneity in study design, definitions, and endpoints. Quantitative pooling was not performed, since the aim of this review was to provide a narrative synthesis of methodological trends, strengths, and limitations of the available evidence base.

Wherever feasible, results from systematic reviews or meta-analyses were prioritized over single-center experience to depict a broader representation of the literature. Primary studies were cross-checked for duplicate datasets to avoid duplication of evidence [8,9].

Given the heterogeneity in study design, populations, and outcome definitions, the review conclusions must be considered interpretive and hypothesis-generating rather than confirmatory, because no statistical pooling, meta-analytic inference, or effect-size comparison was performed.

### 2.4. Quality Assessment and Limitations

As this review did not employ formal meta-analytic methods, no pooled statistical test of bias (e.g., Egger’s or funnel plot) was conducted. Instead, all studies included were evaluated qualitatively for methodological rigor, as follows:

- Complication definition and grading (preferably with ISGPS criteria);
- Completeness of outcome reporting (90-day morbidity/mortality, POPF);
- Presence of risk-adjusted or propensity-matched analyses.

The primary weaknesses of this approach are the potential publication bias, between-study heterogeneity, and over-representation of high-volume expert centers. These weaknesses are noted and are dealt with in the later sections of the review.

## 3. Historical Development and Robotic Technology

The evolution of PD follows the overall trajectory of HPB surgery—from pioneering open resections to more sophisticated minimally invasive approaches. The original Whipple procedure, first described by Allen O. Whipple in 1935, was a milestone for pancreatic surgery [9]. Over the next several decades, improvements in perioperative care, vascular reconstruction, and POPF management dramatically reduced mortality, but morbidity remained high even in specialized centers [10].

### 3.1. Transition to Minimally Invasive Surgery

Gagner and Pomp initially described the LPD in 1994, demonstrating the feasibility of performing this complex operation with minimal access [11]. However, widespread application of LPD was hindered by inherent limitations of laparoscopy, such as two-dimensional vision, rigid instrumentation, the fulcrum effect, and the difficulty of precise intracorporeal suturing required for pancreatic and biliary anastomoses [12]. As a result, LPD remained confined to a few specialist centers, with lengthy learning curves and high conversion rates.

### 3.2. Emergence of Robotic Platforms

The advent of robot-assisted surgery in the early 2000s revolutionized minimally invasive HPB surgery. The da Vinci^®^ Surgical System (Intuitive Surgical, Sunnyvale, CA, USA), approved by the FDA in 2000, offered three-dimensional magnified vision, seven degrees of freedom, tremor filtration, and improved ergonomics for the operating surgeon [13]. These technological innovations bypassed most of the drawbacks of conventional laparoscopy and opened up new possibilities for complex procedures such as PD.

In 2003, Giulianotti et al. described the initial RPD, demonstrating proof of concept that a technically demanding procedure like this could be completed totally robotically [14]. Additional case reports and small series confirmed the feasibility and safety of RPD, but operative times were initially long and learning curves steep [15].

Several authors have pointed out that, in contrast to the laparoscopic approach, the shift from OPD to a robotic technique seems more intuitive and less technically disruptive for surgeons trained in complex open HPB procedures [11,12,14,15]. This advantage is afforded by key technological characteristics of the robotic platform, such as three-dimensional high-definition visualization, enhanced depth perception, articulated EndoWrist^®^ instruments with seven degrees of freedom, tremor filtration, and superior ergonomics, which closely mimic the hand–eye coordination and the suturing mechanics of open surgery [13,14].

On the other hand, the laparoscopic approach has several psychomotor and perceptual limitations that surgeons must overcome, such as the fulcrum effect, rigid long-shaft instruments, limited triangulation, restricted freedom of movement, and two-dimensional imaging, all adding significantly to the difficulty in performing complex biliary and pancreatic anastomoses [11,12]. Until recently, these challenges have hindered the wider diffusion of LPD despite early demonstrations of feasibility [11,12].

Consequently, various high-volume centers have reported successful implementation of RPD programs even among surgeons with limited prior laparoscopic experience. Such programs favor a direct transition from open to robotic surgery through structured modular curricula, dual-console mentoring, simulation-based training, and stepwise progression models [15,16,17,18]. This evolution partly explains the faster global diffusion of robotic pancreaticoduodenectomy compared with its laparoscopic counterpart and underlines the role of robotics as a more accessible gateway to minimally invasive pancreatic surgery for traditionally open-trained HPB surgeons [14,15].

### 3.3. Expansion and Maturation of RPD

**Figure 1:**
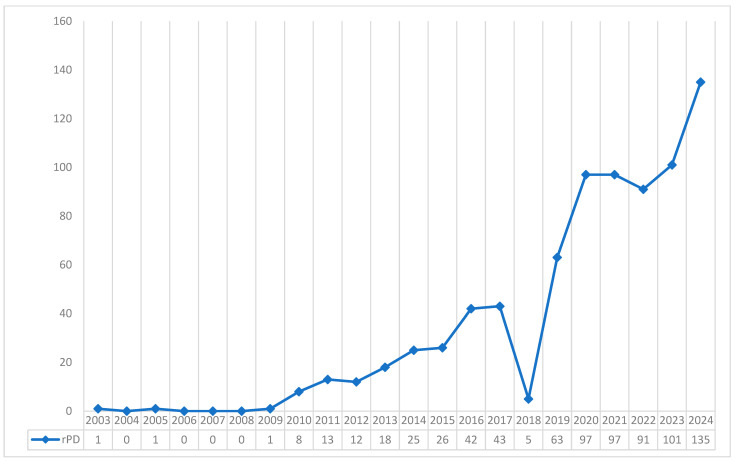
Increase in publications on rPDs.

The past decade has witnessed cumulative experience and technical modifications that permit a steep learning curve for RPD programs worldwide. Large single-institution and multicentre reports from the United States, Europe, and Asia have demonstrated reproducible results comparable to open or laparoscopic PD in high-volume centers [19]. Figure 1 highlights how the number of articles published on PubMed has increased exponentially when searching for the keywords ‘Robotic Pancreaticoduodenectomy’.

Meta-analyses currently show RPD to be associated with reduced blood loss and length of hospitalization, with equivalent mortality and oncologic completeness compared to traditional approaches [20]. Importantly, with increasing cumulative operative experience, the prolonged early operative times routinely decrease significantly, reflecting the procedural learning curve and familiarization with technology [15].

### 3.4. Technological Advances

Robotic technology itself has also evolved considerably from the original da Vinci S and Si platforms. The introduction of the da Vinci Xi platform provided increased arm flexibility, more streamlined docking, and a more ergonomic trocar configuration, allowing multi-quadrant access necessary for PD [21]. More recently, next-generation robotic systems such as Hugo™ RAS (Medtronic), Versius™ (CMR Surgical), and SS Innovations’ SSi Mantra™ provide enhanced modularity, portability, and potentially reduced costs, which can facilitate greater access to robotic surgery and democratize it beyond high-volume centers [22].

Besides the da Vinci^®^ Si and Xi platforms, which have been the backbone of the development of robotic pancreatic surgery over the last decade [21,22], a new generation of robotic systems has recently emerged to further improve ergonomics, instrument versatility, and intraoperative safety. Within this context, the da Vinci^®^ 5 platform represents the latest evolution of the Intuitive Surgical ecosystem, with the introduction of refined haptic feedback technology devised to partially overcome one of the historical limitations of robotic systems, that is, the absence of tactile perception [13].

Preliminary reports indicate that this advanced haptic interface may enhance tissue handling, vascular dissection accuracy, and needle control, especially in fragile phases such as pancreatic transection and duct-to-mucosa suturing, although formal outcome-based evidence is still scarce considering the early stage of its adoption [22]. In concert with Intuitive’s next-generation development, a new crop of up-and-coming challengers such as Hugo™ RAS from Medtronic, Versius™ by CMR Surgical, and SSi Mantra™ have introduced modular architectures, portable consoles, and cost-containment models to further widen the accessibility of robotic pancreatic surgery worldwide [22].

Overall, the integration of next-generation systems with advanced sensory feedback, digital integration, and interoperable imaging technologies is likely to further improve procedural reproducibility, training, and broader dissemination of RPD in the coming years [22].

### 3.5. Current Status

By 2025, RPD will represent one of the most advanced instances of minimally invasive HPB surgery. Although still concentrated in high-volume centers with formal robotic programs, cumulative global data now comprise thousands of cases [23]. With increasing evidence demonstrating equivalent safety and oncologic outcomes, the robotic approach is now considered not merely an experimental alternative but rather an acceptable option within selected institutions possessing the necessary expertise, infrastructure, and multidisciplinary support.

## 4. Technical Aspects of Robotic Pancreaticoduodenectomy

The technical execution of RPD integrates the fundamental oncologic principles of the open Whipple procedure with the advantages of robotic technology—enhanced dexterity, tremor filtration, and high-definition three-dimensional vision [24]. Despite these advantages, RPD remains one of the most demanding abdominal procedures, requiring careful patient selection, precise port placement, and mastery of advanced intracorporeal anastomoses.

### 4.1. Patient Positioning and Setup

The patient is placed in a supine split-leg (French) or supine reverse Trendelenburg position with slight left tilt to allow gravitational retraction of the small bowel [25]. The primary surgeon operates from the robotic console, while an experienced bedside assistant manages suction, stapling, and vascular control.

Port placement is critical for ergonomic efficiency. With the da Vinci Xi system, four robotic trocars are typically arranged in a linear or oblique line across the upper abdomen, complemented by one or two assistant ports [24,25]. Docking is usually performed from the patient’s head in a “supracerebral” configuration, minimizing arm collision and optimizing instrument reach across the upper quadrants [24,25].

For detailed visual references of trocar positioning and patient setup, readers are referred to dedicated step-by-step operative atlases and video-based technical guides available in the cited literature [24,25,26,27].

### 4.2. Dissection Phase

The operation begins with an exploratory laparoscopy to exclude metastatic disease. The hepatic flexure of the colon is mobilized, followed by Kocher’s maneuver to expose the inferior vena cava and aorta. The portal triad is identified, and the gastroduodenal artery (GDA) is skeletonized and divided between clips or a vascular stapler. The common hepatic artery and portal vein are then dissected with precise robotic motion that allows atraumatic handling of small vascular branches [24,25].

The pancreatic neck is identified over the portal vein and divided using ultrasonic shears or monopolar scissors, while the pancreatic duct is isolated for subsequent anastomosis. The uncinate process is detached from the superior mesenteric vein and artery (SMV/SMA) using a combination of bipolar cautery and vessel sealing devices [24,25].

### 4.3. Specimen Extraction and Reconstruction

After completion of the resection phase, the specimen is retrieved via a small Pfannenstiel or periumbilical incision, maintaining oncologic integrity. Reconstruction follows the standard three-anastomosis sequence:

1. Pancreaticojejunostomy (PJ);
2. Hepaticojejunostomy (HJ);
3. Gastrojejunostomy (GJ) or duodenojejunostomy, depending on pylorus preservation.

The pancreaticojejunostomy remains the most technically challenging component. Two predominant robotic techniques are described:

- The duct-to-mucosa anastomosis (Blumgart or modified Kakita technique), utilizing 5-0 or 6-0 monofilament sutures placed with precise robotic wrist articulation [24,26];
- The invagination (dunking) technique, suitable for soft glands or small ducts, in which the pancreatic stump is telescoped into the jejunal lumen and secured with a double-layer suture [27].

The hepaticojejunostomy is performed with interrupted or continuous absorbable sutures (typically 4-0 or 5-0 PDS). Robotic articulation facilitates accurate placement of posterior wall stitches, which are traditionally the most demanding in open or laparoscopic settings [24,25,27].

Gastrojejunostomy (or duodenojejunostomy) can be completed robotically with a stapled or hand-sewn approach, depending on the surgeon’s preference. Robotic staplers integrated with the Xi system allow for precise angle control and consistent staple line formation [24].

Pancreatic duct management is a crucial determinant of postoperative outcome after reconstruction. Internal stents and external transanastomotic drainage have both been employed in the attempt at protecting the PJ, thereby ensuring controlled ductal outflow, especially in the face of soft pancreas texture or small-caliber ducts. Internal stents—whether resorbable or silicone devices—may decrease abdominal wall exposure to a foreign body, enhance patient comfort, and potentially facilitate postoperative management; however, concerns remain regarding the risk of intraluminal dislocation and impaired ductal clearance [24,26,27].

On the other hand, external drainage through percutaneous transanastomotic stents has been related to better monitoring of pancreatic secretion and earlier identification of clinically relevant POPF but may increase discomfort for the patient and require additional steps of postoperative management [26,27]. Evidence is still heterogeneous, and no standardized consensus has established the superiority of either approach to date; thus, duct management should be individually tailored based on intraoperative tissue characteristics, surgeon experience, and institutional protocols.

Aside from percutaneously placed external stents, a common alternative is the intraoperatively placed external intrapancreatic transanastomotic stent inserted through the pancreatic stump and across the pancreaticojejunostomy with its distal end externalized through the abdominal wall. Several randomized trials and meta-analyses report that externalized transanastomotic stents are associated with a lower incidence of postoperative pancreatic fistula (POPF) compared with no stent or internal stents: a pooled analysis reported a marked reduction in POPF (OR ≈ 0.34) and shorter length of stay with external stents [28]. Subgroup/meta-analytic data have similarly shown a reduced risk of clinically relevant POPF with external stents (pooled RR ≈ 0.61 in some analyses) [16]. Several randomized and multicenter trials have directly compared external versus internal transanastomotic drainage, with heterogeneous results: while some single RCTs, for example, Tani et al., did not demonstrate a clear superiority of one approach in all-comers, larger pooled and subsequent trials support a benefit of external drainage, particularly in high-risk situations (soft gland, small duct) [29,30]. A recent randomized phase-3 trial and subsequent technical reviews reinforce the role of externalized stents as a useful mitigation strategy in high-risk anastomoses, though stentrelated issues (malfunction, dislodgement, patient discomfort, need for management of the external limb) are not negligible—transanastomotic stent malfunction was reported in series to occur in a substantial minority of cases, for example, ~36% malfunction in a multicenter report [31].

Taken together, the evidence suggests that intraoperative external intrapancreatic transanastomotic stents should be explicitly differentiated from percutaneously placed external drains: the two techniques differ in timing, technical logistics, physiologic rationale, and risk–benefit profile, and external transanastomotic stents appear most beneficial in patients at high risk for POPF. Consistent with the body of this review, these points align with the reconstruction and stent-management discussion already included in the manuscript; see Section 4.3 and references therein.

### 4.4. Vascular Resection and Reconstruction

One of the major advantages of RPD is the enhanced precision during vascular dissection. Select high-volume centers have reported successful robotic portal vein and SMV resections with primary end-to-end anastomosis or patch reconstruction [25]. The 3D visualization and stable magnification permit safe manipulation near critical vascular structures, while the surgeon’s console control reduces tremor and fatigue.

However, RPD with vascular resection remains controversial. A recent analysis by Napoli et al. (2024) found that while robotic vascular resection is feasible, it is associated with significantly longer operative time and higher resource utilization without a clear survival advantage [32]. Thus, vascular RPD should be limited to centers with established robotic HPB expertise and multidisciplinary support.

### 4.5. Operative Time, Learning Curve, and Standardization

Early RPD series reported median operative times exceeding 600 min [15,19,20]. However, with structured training, simulation, and case standardization, this duration can decrease below 400 min after approximately 40–60 cases, as shown by Zureikat et al. [15].

Procedure standardization has become essential to improving reproducibility. The U.S. Robotic HPB Consortium proposed a stepwise, modular training pathway that incorporates simulation, dual-console mentoring, and proctored cases to shorten the learning curve and improve outcomes [33]. Similar structured programs have been adopted in Europe and Asia, emphasizing credentialing and video-based performance assessment.

### 4.6. Innovations and Intraoperative Guidance

Recent innovations have focused on intraoperative imaging and navigation. The integration of indocyanine green (ICG) fluorescence aids in the identification of biliary anatomy and the assessment of anastomotic perfusion, potentially reducing the risk of bile leaks and ischemic complications [34].

Robotic integration with augmented reality (AR) and near-infrared (NIR) fluorescence is under investigation for enhanced orientation during vascular dissection and lymphadenectomy [35]. Furthermore, the combination of RPD with real-time intraoperative ultrasound enables precise localization of the pancreatic duct and major vessels, supporting safe resection margins [36].

## 5. Perioperative and Oncologic Outcomes

The evaluation of perioperative and oncologic outcomes is crucial for defining the clinical value of RPD. Although initial adoption was confined to high-volume centers, recent multicentre data and meta-analyses have allowed for a more objective comparison between RPD, open pancreaticoduodenectomy (OPD), and LPD. Overall, RPD demonstrates comparable safety, reduced intraoperative blood loss, and similar oncologic efficacy when performed by experienced surgeons.

Interpretation of these results must consider substantial heterogeneity across studies. High-volume robotic centers tend to report lower morbidity, shorter LOS, and reduced conversion rates, introducing a volume-related bias. In addition, definitions of POPF, DGE, and PPH vary in different studies, with inconsistent adoption of ISGPS criteria. This limits the comparability of published series and partially explains the variability in reported outcomes.

### 5.1. Operative Time and Intraoperative Metrics

Early single-institution experiences reported median operative times exceeding 600 min, primarily due to docking, instrument exchange, and intracorporeal suturing complexity [15]. However, subsequent data indicate that as procedural experience increases, operative duration decreases significantly to around 400 min, approximating that of LPD [37]. In their paper, DeLaura et al. identified a clear learning curve plateau after approximately 40–60 procedures, beyond which operative times, blood loss, and conversion rates stabilized [38].

A recent meta-analysis confirmed that RPD was associated with significantly lower intraoperative blood loss compared to OPD, while operative time was longer by approximately 70 min [39].

### 5.2. Conversion Rates

Conversion from robotic to open surgery occurs in 5–10% of cases, typically due to bleeding, dense adhesions, or difficult vascular dissection [32,34]. Risk factors include high BMI, soft pancreatic texture, and peri-arterial tumor involvement. The robotic approach may reduce conversion compared with pure laparoscopy, given the enhanced dexterity and visualization [37].

These percentages derive predominantly from high-volume robotic HPB centers, where standardized workflows, mentorship, and cumulative experience significantly reduce intraoperative uncertainty [37,39]. In lower-volume settings, conversion rates may be considerably higher, as reported in national and registry-based cohorts, where reduced case frequency, limited staffing expertise, and less structured training pathways have been associated with increased procedural variability [17,40]. These data highlight the importance of institutional commitment, mentoring pathways, and the progressive centralization of robotic pancreatic programs.

### 5.3. Postoperative Morbidity and Mortality

Overall postoperative morbidity after RPD ranges between 35 and 50%, aligning with rates reported for OPD and LPD [37,39].

The most frequent complications are DGE, POPF, and PPH, graded according to the ISGPS criteria. A recent international meta-analysis (Tang et al., 2024 [8]) including >9000 patients reported no statistically significant difference in the rate of clinically relevant POPF (Grade B/C) between RPD and OPD (14.8% vs. 16.2%, *p* = 0.28) [8]. However, RPD was associated with lower overall morbidity (OR 0.78, 95% CI 0.67–0.91) and shorter hospital stay (−2.8 days). Importantly, 90-day mortality remains consistently low (<2%) in high-volume robotic centers, similar to open benchmarks [8,39]

### 5.4. Postoperative Recovery and Hospital Stay

Enhanced recovery after robotic surgery results from minimal tissue trauma, stable pneumoperitoneum, and precise dissection. A multicentre European analysis by Napoli et al. (2021) reported a median length of stay (LOS) of 8 days for RPD, compared with 12 days for OPD (*p* < 0.001) [5]. Patients undergoing RPD also demonstrated earlier return of bowel function and faster resumption of oral diet, facilitating implementation of ERAS (Enhanced Recovery After Surgery) protocols [41].

Table 1 summarizes the most important meta-analyses and comparative studies from 2016 to 2025.

### 5.5. Oncologic Outcomes

Available evidence suggests that oncologic outcomes, defined by R0 resection rate, lymph node yield, and survival, may be comparable with open surgery when performed in expert hands.

In a single-institution retrospective review of 456 patients who received RPD or OPD, Girgis et al. reported similar rates of R0 resection but higher lymph node harvest (21.47% vs. 21.72%), (31.9  ±  12.2 vs. 25.9  ±  11.1; *p*  <  0.0001) [18].

Similarly, Nassour et al. (2018) [42] showed that there was no difference in median overall survival between RPD (22.7 months) and LPD (20.7 months). RPD was also not associated with inferior overall survival [hazard ratio (HR)  =  0.99; 95% CI 0.75–1] [43].

Long-term follow-up data remain limited, but they are encouraging.

### 5.6. Cost Analysis and Resource Utilization

Economic evaluation remains highly heterogeneous. Costs vary substantially according to procedure volume, hospital reimbursement models, and country-specific healthcare systems. Cost neutrality reports are typically from high-volume centers where shorter LOS offsets instrumentation costs, whereas low- and medium-volume hospitals show significantly higher total expenses. Comparability across studies is limited by the lack of standardized cost-reporting templates.

Another limitation in cost literature is that most analyses report only direct operative costs and exclude indirect costs such as readmissions, long-term complications, and societal impact. Moreover, few studies stratify costs by learning curve phases, despite strong evidence that early cases are significantly more expensive than those performed after proficiency is reached.”

A 2021 single-institution cost-analysis by Rosemurgy et al. found that total direct cost for RPD was approximately 36% higher than OPD (USD 31,389 vs. 23,132) (*p* = 0.04) [44], while Aguayo et al. showed that index costs were non significantly different ($51,956 vs. $47,296, *p*  =  0.28) [45].

## 6. Training and Learning Curve, and Future Perspectives

The safe and effective implementation of RPD requires a structured approach to training and credentialing, given the technical complexity of the procedure. The learning curve is a critical determinant of perioperative outcomes, oncologic quality, and resource utilization. Moreover, ongoing technological innovations are poised to reshape both training paradigms and clinical practice.

A number of studies point to the merit of step-wise, modular training programs for RPD. These training programs, designed by the U.S. Robotic HPB Consortium and other high-volume programs, include the following:

1. Dry-lab simulation-based and virtual reality-based training to familiarize surgeons with robotic console ergonomics and instrument handling;
2. Proctored dual-console procedures, enabling direct supervision by a skilled robotic surgeon while leaving the trainee to carry out discrete operative steps;
3. Video-based testing and measurement of performance, enabling objective assessment of mastery of skills and identification of technical shortcomings [46].

Evidence further indicates that formal training reduces operative time, conversion rates, and postoperative morbidity early after surgery by a significant amount. Conversely, surgeons attempting RPD without formal mentorship have longer learning curves and increased perioperative morbidity [42].

The RPD learning curve has been the subject of thorough analysis. Preukschas et al. demonstrated that 40–60 cases are required to master operative time, blood loss, and conversion rates, while optimal postoperative outcomes and maximum reduced complication rates can take a maximum of 80–100 cases [47].

Stepwise improvement is confirmed by learning curve analysis in the following features:

- Operative efficiency (instrument exchange and time);
- Technical precision (vascular dissection and PJ);
- Clinical outcomes (POPF, DGE, LOS, morbidity).

Institutional high-volume experience remains a paramount determinant of successful learning. Low-volume centers are associated with higher rates of conversion and complications and emphasize the need for centralized robotic HPB programs [40].

Professional societies, including the International Hepato-Pancreato-Biliary Association (IHPBA), recommend the following:

- Completion of a high-volume HPB fellowship;
- Stepwise supervised robotic training;
- Minimum threshold cases for independent practice (usually ≥40–50 RPDs under supervision);
- Regular review with structured video review and objective metrics [17].

Institutions are encouraged to maintain centralized robotic HPB programs with multidisciplinary support to optimize outcomes and patient safety.

Emerging technologies are transforming RPD education. They comprise the following features:

- Immersive VR and AR simulators that enable rehearsal of procedural steps in virtual environments and intraoperative scenario management;
- Artificial intelligence (AI) video-assisted reviews enabling an objective assessment of suture quality, dissection plane identification, and optimization of critical steps;
- 3D-printed models of the pancreas for anastomoses and vascular reconstruction practice.

These technologies reduce the volume of first-life cases necessary to achieve proficiency and provide a safer environment for trainee surgeons.

Several strategies are on the horizon to break through existing barriers and expand the role of RPD:

1. Technological advancement
  - Next-generation robotic systems with haptic feedback, smaller footprints, and low-cost consumables are on the horizon.
  - Augmented reality (AR) integration, indocyanine green (ICG) fluorescence, and AI-guided intraoperative assistance can improve safety and precision.
2. Augmented training pathways
  - Simulation-based training, two-console mentorship, and stepwise modular curricula can minimize learning curves and improve reproducibility.
3. Centralization and volume-driven care
  - High-volume center consolidation of RPD assures higher quality results, streamlines complex case management, and allows for systematic long-term oncologic efficacy assessment.
4. Clinical research and data sharing
  - Future large randomized trials and prospective multicenter registries ought to be conducted to determine the following:
    ○ Long-term oncologic equivalence of OPD and RPD.
    ○ Cost-effectiveness and resource utilization.
    ○ Vascular reconstruction and outcomes of complex pancreatic resections.
5. Tele-mentoring and remote surgery
  - Tele-mentoring and remote supervision can enhance accessibility to robotic expertise in low-volume centers without jeopardizing safety standards.

Overall, RPD represents a paradigm shift in pancreatic surgery, wedging minimally invasive benefits to oncologic rigor. Long-term success of RPD will depend on structured training, technological innovation, and centralization of experience.

However, despite enhanced dexterity and visualization, RPD is still constrained by several technical factors:

- Longer operative times, particularly in early learning curve patients, encourage anesthesia exposure and operative fatigue.
- Poor tactile feedback, which can impair judgment during vascular dissection and manipulation of pancreatic parenchyma, leads to complete reliance on visual data and increases the imaging necessary.
- Rare vascular reconstruction, like portal vein or superior mesenteric vein resection, is challenging technically and ideally recommended in high-volume centers.

Patient-specific variables, such as elevated BMI, history of previous upper abdominal surgery, or advanced local disease, further contribute to operative complexity and risk of conversion.

The expense of RPD is currently still a significant limitation to widespread adoption. The increased initial capital expenditures on robotic platforms, upkeep, and consumables over conventional laparoscopic or open surgery are both anecdotally described and published in some research. While some studies suggest reduced hospital stay and reduced complications might offset these expenses, specific cost-effectiveness data are still limited and institution-specific by volume and patient selection.

Moreover, while current evidence suggests RPD provides comparable R0 resection rates and lymph node harvest as OPD, long-term survival experience is sparse. In borderline resectable or locally advanced cancers requiring vascular reconstruction, likewise, solid comparative long-term oncologic results are unavailable, and the relative benefit of RPD in such a circumstance remains under investigation.

## 7. Limitations

This review and the evidence currently available on RPD present several limitations that must be acknowledged.

Most of the data published originates from retrospective observational studies, usually without standardized complication grading or homogeneous oncologic endpoints [8,32,34]. Randomized controlled trials focused on comparing robotic, laparoscopic, and open approaches are still lacking; therefore, this reduces the strength of causal inference.

Moreover, the majority of published series emanate from high-volume referral centers, introducing the possibility of selection bias and limiting generalizability to smaller institutions or early adoption programs [15,18,32]. Of course, publication bias may also be a factor, as reports more often reflect successful experiences from expert surgeons and established teams rather than the full spectrum of outcomes.

Differences in postoperative outcome definitions across studies, including heterogeneous adoption of ISGPS criteria, limit comparability and preclude formal harmonization.

Prospective multicenter registries and well-designed randomized studies are, therefore, needed to establish long-term oncologic equivalence, cost-effectiveness, and applicability across different health–system environments.

## 8. Conclusions

RPD is a significant advance in minimally invasive hepatopancreatobiliary surgery that combines the oncologic principles of open pancreaticoduodenectomy and the technical advantages of robotic technology, including greater dexterity, tremor filtering, and high-definition three-dimensional visualization.

Evidence to date demonstrates that, when performed in high-volume centers by experienced surgeons, RPD offers the following advantages:

- Equivalent oncologic results (R0 resection rates, lymph node yield, and survival) to open surgery;
- Less intraoperative blood loss and more rapid postoperative recovery, with shorter length of stay and earlier return of gastrointestinal function;
- Capacity for complex reconstructions, such as selected vascular resections, in skilled centers.

Despite such advantages, there are still some limitations: the procedure is technically demanding, with a steep learning curve, high costs, and requires appropriate patient selection. Formal training programs, simulation teaching, and mentorship, along with centralization in high-volume centers, are required in order to optimize outcomes and ensure patient safety.

In the future, ongoing advances in technology—including next-generation robot systems, augmented reality, intraoperative fluorescence imaging, and artificial intelligence-based navigation—will render RPD increasingly accurate, reproducible, and available. These technologies could potentially reduce the learning curve and expand the spectrum of tumors to which RPD can be delivered, including more complex and borderline resectable tumors.

In conclusion, RPD is currently considered a potentially safe and effective minimally invasive procedure in well-selected patients. Extensive adoption will depend on continuous technology advancement, adequate training, and multicenter evidence confirming perioperative and long-term oncologic benefits. As technology evolves, RPD is going to be an integral element of modern pancreatic surgery, combining minimally invasive advantages with unshelved oncologic stringency.

## Figures and Tables

**Table 1:** Most important meta-analyses and comparative studies from 2016 to 2025. RPD: robotic pancreaticoduodenectomy; OPD: open pancreaticoduodenectomy; LOS: length of stay.

Author (Year)	Study type	N° of Patients	Comparison	Main findings	Conclusion
Tang et al., 2024 [8]	Meta-analysis	>9000	RPD vs. OPD	Lower morbidity, shorter LOS, similar R0 rate	RPD is safe and effective
Fu et al., 2022 [39]	Meta-analysis	~6000	RPD vs. OPD	Less blood loss, similar oncologic outcomes	RPD advantageous perioperatively
Zureikat et al., 2016 [15]	Multicenter study	700	RPD vs. OPD	Equivalent R0 and survival; shorter LOS	Learning curve crucial
Napoli et al., 2021 [5]	Propensity-matched analysis	300	RPD vs. OPD	Reduced LOS, equivalent mortality	RPD matures in expert centers
Girgis et al., 2019 [18]	Single-institution retrospective	456	RPD vs. OPD	Higher lymph node yield, similar R0	Comparable oncologic outcomes
Jones et al., 2024 [42]	International multicenter	635	RPD vs. OPD	Improved efficiency after 60 cases	Supports structured training

## Data Availability

No data were created.
